# Diagnostic accuracy in field conditions of the sickle SCAN® rapid test for sickle cell disease among children and adults in two West African settings: the DREPATEST study

**DOI:** 10.1186/s12878-018-0120-5

**Published:** 2018-09-17

**Authors:** Akueté Yvon Segbena, Aldiouma Guindo, Romain Buono, Irénée Kueviakoe, Dapa A. Diallo, Gregory Guernec, Mouhoudine Yerima, Pierre Guindo, Emilie Lauressergues, Aude Mondeilh, Valentina Picot, Valériane Leroy

**Affiliations:** 1 CHU Campus, Lomé, Togo; 2 Centre de Recherche et Lutte contre la Drépanocytose, 03 BP: 186 BKO 03, Point G, Commune III, Bamako, Mali; 3 Inserm UMR 1027, Epidémiologie et analyses en santé publique : risques, maladies chroniques et handicaps, Université Paul Sabatier Toulouse 3, Faculté de Médecine Purpan, 37 Allées Jules Guesde, 31073 Toulouse Cedex 7, France; 4 Département de Santé Publique, Université de Lomé, Lome, Togo; 5 Pierre Fabre Foundation, Lavaur, France; 6 Mérieux Foundation, Lyon, France

**Keywords:** Sickle cell disease, Diagnosis, Rapid diagnosis test, Sensitivity, Specificity, Performances, Africa

## Abstract

**Background:**

Sickle cell disease (SCD) accounts for 5% of mortality in African children aged < 5 years. Improving the care management and quality of life of patients with SCD requires a reliable diagnosis in resource-limited settings. We assessed the diagnostic accuracy of the rapid Sickle SCAN® point-of-care (POC) test for SCD used in field conditions in two West-African countries.

**Methods:**

We conducted a case-control study in Bamako (Mali) and Lomé (Togo). Known cases of sickle cell disease (HbSS, HbSC), trait (HbAS), HbC heterozygotes (HbAC) and homozygous (HbCC), aged ≥6 months were compared to Controls (HbAA), recruited by convenience. All subjects received both an index rapid POC test and a gold standard (high-performance liquid chromatography in Bamako; capillary electrophoresis in Lomé). Personnel conducting tests were blinded from subjects’ SCD status. Sensitivity and specificity were calculated for each phenotype. Practicality was assessed by local healthcare professionals familiar with national diagnostic methods and their associated constraints.

**Results:**

In Togo, 209 Cases (45 HbAS, 39 HbAC, 41 HbSS, 44 HbSC and 40 HbCC phenotypes) were compared to 86 Controls (HbAA). 100% sensitivity and specificity were observed for AA Controls and HbCC cases. Estimated sensitivity was 97.7% [95% confidence interval: 88.0–99.9], 97.6% [87.1–99.9%], 95.6% [84.8–99.5%], and 94.9% [82.7–99.4], for HbSC, HbSS, HbAS, and HbAC, respectively. Specificity exceeded 99.2% for all phenotypes. Among 160 cases and 80 controls in Mali, rapid testing was 100% sensitive and specific. Rapid testing was well accepted by local healthcare professionals.

**Conclusion:**

Rapid POC testing is 100% accurate for homozygote healthy people and excellent (Togo) or perfect (Mali) for sickle cell trait and disease patients. In addition to its comparable diagnostic performance, this test is cheaper, easier to implement, and logistically more convenient than the current standard diagnostic methods in use. Its predictive value indicators and diagnostic accuracy in newborns should be further evaluated prior to implementation in large-scale screening programs in resource-limited settings where SCD is prevalent.

## Background

Sickle Cell Disease (SCD) is the most common inherited blood disorder worldwide, accounting for 5% of the mortality in African children < 5 years of age [1, 2]. In SCD, mutations occurring on the gene encoding for the hemoglobin (Hb) β chain are transmitted in a recessive way, with an impact on the structural, functional and rheological properties of the erythrocyte [3]. 75.5% of the SCD cases worldwide occur in Sub-Saharan Africa. The term “SCD” includes various Hemoglobin variants phenotypes responsible of the characteristic syndrome, but four are predominant in West Africa. The most common and severe form of SCD is the homozygous HbSS phenotype (sickle cell anaemia) inherited from both parents [2, 3]. Other forms of SCD include the compound heterozygotes HbSC disease and HbS/βthalassaemia [4]. HbC homozygotes and heterozygotes are usually well and these are not forms of sickle cell disease [5].

Under-five mortality is high in SCD patients and associated particularly with the HbSS phenotype in sub-Saharan Africa [6]. The cornerstone of care management is based on early diagnosis, ideally neonatal, of SCD to allow prompt parental education and counselling about disease complications, immunization and antibiotic prophylaxis [1, 7].

Thus, improving the care management and quality of life of patients with SCD requires establishing a reliable diagnosis feasible in resource-limited settings [1, 6, 7]. Currently, two tree gold standard approaches are validated for first-level neonatal screening and routine diagnosis: High Performance Liquid Chromatography (HPLC); Capillary Electrophoresis (CE) and Isoelectric focusing (IEF) [8–10]. Their sensitivity and specificity for detection of normal and common abnormal hemoglobin variants are close to perfect [10–12]. Moreover, a quantification of HbA2 and HbF is also possible, allowing the identification of β-thalassemia and hereditary persistence of fetal hemoglobin forms [10–12]. Whatever the technique used for first-level diagnosis, all international guidelines support the need to perform confirmatory tests using another method. However, these methods are neither rapid, nor easy to implement in resource-limited settings, since they require a consequent financial investment with constraints: time, regular restocking of reagent, availability of electricity, mobilization of dedicated and trained technicians, transport and storage of biological samples, and adherence to strict laboratory standards. They also suffer from high loss to follow-up risk, due to the long lag time between sample collection and the delivery of results that could excess 4 to 6 weeks.

Many devices, at different stages of development, among the novel Point-Of-Care (POC) tests for hemoglobin variants, have emerged as potential alternative tools for reliable and simple diagnosis of SCD in developing countries. Among these, we can cite: i) based on the difference of solubility of the HbA and HbS, the microfluidic paper-based analytical devices (μPADs) from Halcyon Biomedical [13]; ii) based on the difference of Red Blood Cells density from an aqueous multiphase system, the Daktari Sickle Cell developed by Daktari Diagnostics, iii) based on lateral flow immunoassay devices, the Hemotype SC from Silver Lake Research Corporation [14] and the Sickle SCAN® from BioMedomics. The latter, a sandwich format chromatographic immunoassay approach developed for the qualitative measurement of HbA, HbS and HbC in whole blood samples, has demonstrated excellent intrinsic performances to detect common Hb variants [13, 15].

As the Sickle SCAN test employs lateral flow immunoassay technology, it has a potential large-scale screening utility in resource-limited settings. The Sickle Scan® POC device has recently been assessed in field conditions in two studies in sub-Saharan Africa, with promising results [16, 17]. However, none of these studies provided information regarding the climate conditions. The aim of this study is to assess the diagnostic accuracy (sensitivity and specificity) of the Sickle SCAN® POC test and its acceptability to persons conducting the tests under field conditions (temperature and relative humidity) in children older than 6 months of age and adults, in Lomé (Togo) and Bamako (Mali), West Africa.

## Methods

### Study design

DREPATEST was a multicenter diagnosis accuracy study based on a case-control design (1 case for 2 controls), stratified on the two clinical sites (Bamako, Mali, and Lomé, Togo), and reported according to the Standard for Reporting Diagnosis Accuracy [18]. The study was conducted from May 23, 2016 to October 16, 2016.

### Ethics approval and consent to participate

Approvals from ethical committees in both Togo (« Comité d’Ethique de la Recherche en Santé du Togo ») and Mali (“Comité d’Ethique Institutionnel du CRLD du Mali”) were obtained in April and May 2016, respectively. Individual consent was obtained from each participant. The manuscript does not contain any individual person’s identifiable data or information.

### Population

In each site, we selected a convenience samples of patients with SCD (HbSS and HbSC), with sickle cell trait (HbAS) and/or patients homozygous and heterozygous for HbC (HbCC, HbAC), and compared these each to two healthy controls (HbAA) at the same site. Due to their high prevalence in Togo, patients homozygous for HbC (HbCC) can only be recruited in CHU Campus in Lomé.

Subjects were recruited either during their initial diagnosis visit or during a follow-up visit at the Centre de Recherche et de Lutte contre la Drépanocytose (CRLD) of Bamako (Mali), or at the Unité d’Hématologie Clinique, Centre Hospitalier Universitaire Campus of Lomé (Togo). Eligibility criteria included: age > 6 months; no blood transfusion received in the last 3 months; absence of severe vaso-occlusive pain episodes; an informed consent form signed either by the participant or his/her guardian (for children). All the subjects were tested using both the index POC test and the reference standard test in their respective country: HPLC in Bamako [19] and CE in Lomé [9]. In Mali, for all subjects previously diagnosed using the gold standard, a blood drop was collected and used to perform the POC test. Otherwise, in Togo or newly diagnosed Malian subjects, a venipuncture sample collected in EDTA and stored at 4 °C until analysis, providing the blood needed to conduct both the rapid test (immediately) and the standard test (within the 24 h). All the SickleSCAN® tests were performed during the consultation.

### Sample size

PASS 14·0 software was used to calculate the sample size for each assumed parameter of sensitivity and specificity, with a two-sided test following a normal distribution. A total sample size of 240 (which includes at least 158 cases with an abnormal phenotype) achieves 76% power to detect a change in sensitivity from 0.99 to 0.96 using a two-sided binomial test and 19% power to detect a change in specificity from 0.85 to 0.8 using a two-sided binomial test [20]. This corresponds to a total of 280 Togolese subjects, including 200 cases (40 of each of the five Hb abnormal expected phenotypes: HbAS, HbAC, HbSS, HbSC, HbCC) and 80 controls (to be compared with each phenotype), and a sample size in Mali of 240 subjects, including 160 Cases (40 of each of the four Hb phenotypes: HbAS, HbAC, HbSS, HbSC) and 80 controls.

### Procedures

The hemoglobin type was determined using either a high-performance liquid chromatography (D-10 instrument; Bio-Rad) in Mali, or a Capillaris Electrophoresis (MinicapR; Sebia) in Togo to perform the reference test. Sickle SCAN™ is a qualitative lateral flow immunoassay that indicates the presence of HbA, HbS, and HbC in whole blood samples using the sandwich format chromatographic immunoassay approach with colorimetric detector nanoparticles conjugated to antibodies. The rapid test was performed according to the BioMedomics protocol, with 5 μl of blood from the finger or from the venipuncture added to the prefilled buffer solution. After mixing by inverting the bottle tree times, three drops were discarded and five other drops were dropped to the testing cartridge. Five minutes later, the result was read. The rapid test manufacturer-recommended temperature ranges for storage is between 2 °C and 30 °C, while there was no manufacturer-recommended humidity ranges recommended.

All POC tests have been assessed under field conditions including the specific conditions of expedition by plane, and storage to according to the local customs, either in Togo or Mali. The POC tests were stored under local temperatures and humidity conditions. These environmental parameters were recorded alongside the reading of test results, 5 min after a blood sample was obtained and loaded. A photo was taken of each test result, thereby allowing for a subsequent review of findings. Information and clinical care were provided to subjects whose SCD or trait were first diagnosed with the reference method. All POC providers were trained to perform and interpret the Sickle SCAN® tests.

The conduct of this study can be likened to a reversed-flow design with a reference standard diagnostic before the POC test [21]. To prevent any interpretation bias, clinical data were not available to personnel conducting the POC.

### Data collection

A two-sided Case Report Form (CRF) linked by an anonymized subject number, served for data collection. The first part, which could only be accessed by the clinical investigators, documented subjects’ socio-demographic and clinical characteristics, including the Hb phenotype obtained by the reference method, if known. The second part contained the results of the POC test and the temperature and humidity parameters recorded in the setting. Data were stored in Microsoft© Access 2013, specific queries guaranteed the detection of potential missing, inconsistent or duplicate data.

The acceptability of the rapid test to health care workers was assessed using a self-administered qualitative and descriptive survey, collecting: the perception of the user manual’s clarity; perception of the time to obtain the result, and the complexity level felt to interpret the result.

### Statistical analysis plan

Each investigation site’s data were analyzed distinctly using R 3·2·5. Socio-demographic, medical, and site characteristics were described and compared between Hb phenotypes (HbSS, HbSC, HbCC, HbAS, HbAC) and controls (HbAA). Results are expressed with numbers and percentages for categorical variables, or with medians and interquartile ranges for continuous variables. Comparative analysis was conducted between the sum of cases and controls, using Fisher’s exact test or Chi-Square tests for percentages and t-tests or F-tests for means. A type 1 error risk α equal to 0·05 was chosen for the set. For each Hb phenotype, sensitivity and specificity were evaluated with a 95% confidence interval assuming a binomial distribution. We used a logistic regression model to predict the Hb phenotype obtained by the reference method, including the SickleSCAN® test outcome as the main explanatory variable. Other variables included: age (years); sex; intake of antimalarial therapy; iron and vitamin B9 supplementation; ambient temperature (°C); and relative humidity (%). A multivariate analysis was conducted with the variables having a *P*-value < 0·20 in the univariate models. Continuous covariates were categorized when linearity assumption could not be checked before being integrated into the logistic models. Fitting of the adjusted final model was made using a backward elimination approach. Adjusted Odds Ratio (aOR) with their 95% confidence intervals (95% CI) were calculated for each variable. Receiver Operating Characteristic (ROC) curves were constructed and Areas Under the Curve (AUC) and their 95% CI were examined to compare the ability of different models to predict each Hb phenotype.

## Results

### Lomé study: POC versus CE

**Fig. 1:**
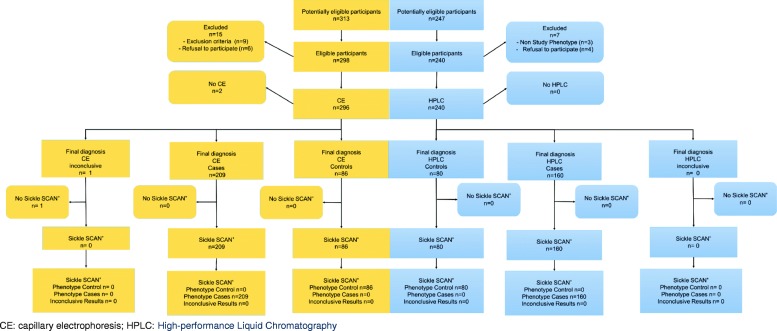
Flow Chart of participants in Lomé (Togo), Mai-June 2016, (*n* = 295) (On the left side) and in Bamako (Mali), October 2016, (*n* = 240) (On the right side)

From May 23rd to June 30th 2016, 313 subjects were eligible in Togo (Fig. 1). Nine subjects failed to meet the required criteria, and six persons declined to participate. In addition, two blood samples were of insufficient quality to be used with the reference test. Of the 296 samples tested using the reference method (CE), only one gave an inconclusive result. Overall, the study was conducted among the remaining 295 subjects (94%): 209 abnormal phenotypes (45 AS, 39 AC, 41 SS, 44 SC and 40 CC phenotypes), and 86 Controls (AA).

There were significantly more men (73.3%) among abnormal phenotypes than in controls (58.4%) but no other statistically significant differences were noted (Table 1). Three children were younger than 1 year at inclusion. The POC devices have been stored during 60 to 73 days at a median ambient temperature of 29.5 degrees (range: 28.4 °C – 35 °C) and a median humidity of 72% (range: 62–77%).

**Table 1:** Baseline characteristics between sickle cell anaemia and trait cases and controls in Lomé (Togo) and Bamako (Mali)

Haemoglobin Phenotype	Lomé (Togo), reference = Capillary electrophoresis (*n* = 295)							Bamako (Mali), Gold Standard = HPLC (*n* = 240)					
	Controls	Cases p^a^	AS	AC	SS	SC	CC	Controls	Cases p^a^	AS	AC	SS	SC
	*N* = 86 (%)	*N* = 209 (%)	*N* = 45 (%)	*N* = 39 (%)	*N* = 41 (%)	*N* = 44 (%)	*N* = 40 (%)	*N* = 80 (%)	*N* = 240 (%)	*N* = 40 (%)	*N* = 40 (%)	*N* = 40 (%)	*N* = 40 (%)
Age (year)		0·842							0·523				
Median	23·2	23·3	23·4	23·3	11·8	25·7	32·9	25·3	22·4	24·8	17·7	17.1	22·3
Q1-Q3	21·8–26·6	20·0–28·1	21·2–24·6	21·0–26·2	5·8–22·9	15·4–34·1	23·2–40·4	14·9–31·3	11·9–30·2	17·7–31·4	7·1–28·6	10·4–24·7	16·1–28·6
Sex		0·017						1					
Male	63 (73·3)	122 (58·4)	29 (64·4)	26 (66·7)	21 (51·2)	27 (61·4)	19 (47·5)	31 (38·8)	62 (38·8)	14 (35)	16 (40)	13 (32·5)	19 (47·5)
Site conditions													
Temperature (°C)		< 10^−3^							–				
Minimum	28·8	28·4	29·5	28·4	28·8	28·4	28·4	25·6	25·6	–	–	–	–
Q1-Q3	29·5–29·7	28·8–29·7	29·5–30·5	29·5–30·5	28·8–29·5	28·8–28·8	28·4–28·9	25·6	25·6	–	–	–	–
Maximum	30·5	35·0	30·5	35·0	30·5	30·5	30·2	25·6	25·6	–	–	–	–
Relative Humidity (%)		0·36											
Minimum	65	62	65	65	62	62	62	32	32	–	–	–	–
Q1-Q3	70–77	70–76	65–77	65–77	70–75	75–76	72–75	32	32	–	–	–	–
Maximum	77	77	77	77	77	77	76	32	32	–	–	–	–

^a^ Comparison between Controls (AA) and the total of Togolese Cases (AS, AC, SS, SC, CC) or Malian Cases (AS, AC, SS, SC)

Sixty-seven (22.7%) POC tests were performed under ambient temperature above the manufacturer-recommended maximum, ranging from 30·5 °C to 35·0 °C. Ninety-four (31.8%) tests were conducted at a relative humidity greater than 75%, the upper quartile.

**Fig. 2:**
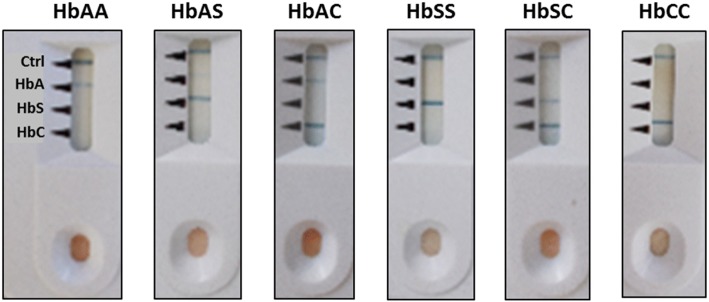
Hemoglobin phenotypes diagnosed with Sickle SCAN

All HbCC subjects and controls (HbAA) were correctly classified using the POC test according to the reference method (Table 2). Figure 2 provides the photos of the POC tests with representative results for each phenotype.

**Table 2:** Aggregate confusion matrix between results achieved by two different reference methods and the Sickle SCAN® device, to determine the haemoglobin phenotype in Lomé (Togo), May–June 2016, (*n* = 295) (On the left side) and in Bamako (Mali), October 2016, (*n* = 240) (On the right side)

		Capillary Electrophoresis									High Performance Liquid Chromatography					
		AA	AS	AC	SS	SC	CC	Total			AA	AS	AC	SS	SC	Total
Sickle SCAN®	AA	86	0	0	0	0	0	86	Sickle SCAN®	AA	80	0	0	0	0	80
	AS	0	43	1	0	0	0	44		AS	0	40	0	0	0	40
	AC	0	1	37	0	1	0	39		AC	0	0	40	0	0	40
	SS	0	1	0	40	0	0	41		SS	0	0	0	40	0	40
	SC	0	0	0	1	43	0	44		SC	0	0	0	0	40	40
	CC	0	0	1	0	0	40	41								
	Total	86	45	39	41	44	40	295		Total	80	40	40	40	40	240

However, over the 295 tests, six misclassifications (2%) were reported among the HbAS, HbAC, HbSS and HbSC cases (Table 2). Out of these six misclassifications, none was observed among the three infants aged less than 12 months and five of them could be related to extreme ambient conditions: one was observed above the manufacturer-recommended temperature (at 30.5 °C) but under normal humidity, four were observed within the manufacturer-recommended temperature ranges, but above 75% of humidity. Two among the observed misclassifications conducted above 75% of humidity were also directly linked to the reading operator misinterpretation. For the last case misclassified observed under “normal ambient conditions”, photos suggest an unclear result, with the visual indicator corresponding to the HbA identification slightly marked when co-associated with other Hb variants (AS, AC).

Sensitivity and specificity estimates (and associated 95% CI) for each Hb phenotype are presented in Table 3. From perfect intrinsic performance for Controls, sensitivity increases between 97.6 and 100% for SCD patients. The lowest sensitivity is observed in HbAS and HbAC trait subjects with 94.9 and 95.6%, respectively. The specificity exceeded 99% in all cases.

**Table 3:** Sensitivity and specificity for each haemoglobin phenotype identified using the reference standard method (Capillary Electrophoresis) in Lomé (Togo), May–June 2016. (*N* = 295)

Haemoglobin phenotype identified by Capillary Electrophoresis	N	Sensitivity (%)	95% CI^a^	Specificity (%)	95% CI^a^
AA	86	100	[93·8–100]	100	[97·4–100]
AS	45	95·6	[84·8–99·5]	99·6	[97·8–99·9]
AC	39	94·9	[82·7–99·4]	99·2	[97·2–99·9]
SS	41	97·6	[87·1–99·9]	99·6	[97·2–99·9]
SC	44	97·7	[88·0–99·9]	99·6	[97·8–99·9]
CC	40	100	[87·1–100]	100	[97·8–100]

^a^ 95% Confidence Intervals (CI) have been computed using the binomial distribution

In the final multivariate analysis, the only variable associated with the reference test outcome was the Hb phenotype determined by the Sickle SCAN® test. Indeed, association between this variable and the variable of interest was perfect for Controls and HbCC Cases and almost perfect for HbAS (aOR = 5353·5; 95%CI [475–60,336]), HbAC (aOR = 2349·5; 95% CI [321·2–17,188·9]), HbSS (aOR = 10,120; 95% CI [620·4–165,067·7]), and HbSC (aOR = 10,750; 95% CI [659·8–175,139·1]).

**Fig. 3:**
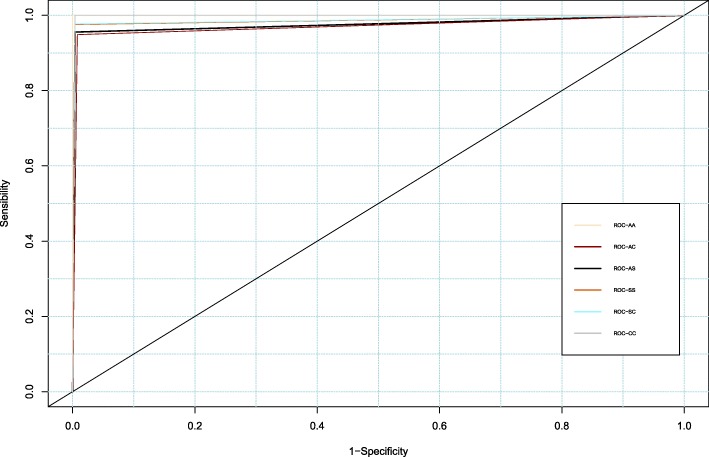
ROC Curves of hemoglobin phenotypes AA, AS, AC, SS, SC and CC in Lomé (Togo), June 2016 (*n* = 295). Area Under the Curve (AUC): AS= 0·976 IC95% [0·945-1], AC= 0·9705 IC95% [0·935-1], AA= 1, SS = 0.9858 IC95% [0·9616-1], SC= 0·9866 IC95% [0·964-1], CC = 0·9866 IC95% [0·994-1]

HbAA discriminating power model is perfect, with a maximum AUC value (Fig. 3). AUC values for the HbAS and HbAC models are 97.6% [95% CI: 94.5–100.0] and 97.1% [95% CI: 93.5–100.0], respectively.

### Bamako: POC versus HPLC

The Mali flow chart is presented in Fig. 1. Among the 247 eligible patients, 98% met the inclusion criteria. There were no differences between cases and controls for the following variables: age, sex, hydroxycarbamide intake and iron supplementation. Vitamin B9 supplementation and antimalarial drug intake were overrepresented in Cases, particularly in HbSS and HbSC patients. Only, three children were younger than 1 year at inclusion. In Mali, all POC tests have been stored during 77 to 119 days and performed in an air-conditioned room at 25.6 °C and 32% of humidity.

For all 240 subjects, the Sickle SCAN® test yielded the same result as the HPLC gold standard, leading to sensitivity and specificity estimates of 100% (Table 2).

### Acceptability to health care professionals

Twenty professionals (17 from Togo and three from Mali), all involved in SCD care, with high levels of clinical laboratory experience and expertise (two hematologists, one pharmacist, one biological engineer and fourteen biological technicians) completed the acceptability questionnaire: 90% perceived the user manual as either “reasonably clear” or “very clear.” The time required to obtain the result was labelled “short” for 95%. All respondents found the interpretation of results to be either “simple” or “very simple.” Each step required to perform the rapid test was described as “simple” or “very simple” to execute for at least 95% of the participants. Specific suggestions included: translating instructions into French; and improving the readability of the “HbA” line in the presence of HbAS or HbAC phenotypes to avoid confusion.

## Discussion

This study is one of the first to evaluate the accuracy of the Sickle SCAN® test under clinical field conditions of a POC test in West Africa using two different gold standards, the HPLC and CE tests. Our study provides new insights into the African operational considerations of using this point-of-care (POC) device under real field conditions that could have been different from those recommended by the manufacturer. This study in different conditions is a necessity given to the difficulties in African settings. We have included operational results with POC device storage and temperature and hygrometry conditions recorded at the time of the testing, the POC performances, as well as a qualitative study to explore the acceptability of the device to a range of health care professionals supposed to use it. In Mali, using the HPLC reference test, sensitivity and specificity were both estimated to be 100%, regardless of the Hb phenotype. For a person expressing a particular Hb phenotype, the likelihood of being diagnosed with an identical Hb phenotype by the Sickle SCAN® test is 100%. This test was performed under favorable conditions of constant temperature and relative humidity, monitored by an air-conditioning system. In Togo, using the CE reference test, the POC test identifies and differentiates HbAA controls and HbCC perfectly. But, compared with the Malian samples, the estimated sensitivity declined for HbAS or HbAC phenotypes, reaching a minimum of 94.9% for HbAC patients. Specificity estimates for all cases were excellent, exceeding 99%. In Togo, among the six misclassifications on a total of 295 samples, five could have occurred under unfavorable ambient conditions (temperature/relative humidity), and one was related to an error of interpretation. Nevertheless, the Sickle SCAN® test allow to perfectly predict HbAA and HbCC phenotypes diagnosed by CE, and prediction is almost perfect for HbAS, HbAC, HbSS, and HbSC (*p* <  10^− 5^). For each model, the power discrimination was not improved after adjustment. Overall, the rate of misclassification is estimated to be 6/535 tests performed, i.e. 1.1%: this rate in a diagnosis strategy could be considered as acceptable, as it will be further confirmed. Thus, we conclude that this POC device is accurate and robust when used under field West-African conditions. But, it also emerges that the sensitivity and specificity of the Togo data with regard to the identification of carriers and patients are lower than the sensitivity and specificity demonstrated for the identification of healthy subjects. In addition, the use of this POC device was acceptable to health care professionals, who found it to be simple, easy to interpret, and yielding results in less than 5 min.

### Validity of the study

All three diagnostic tests – the two comparator reference tests and the and POC index test – were performed and analyzed independently. The index POC test was not a component of the reference test. All tests were administered on a blinded basis; different providers conducted different tests. In addition, no clinical information was provided to persons conducting the Sickle SCAN® test. All subjects were initially diagnosed by a single national reference test, a strict monitoring process insuring control for verification bias. Our CRF contains dedicated space for ambiguous results, and after monitoring, no such results were reported during the study period. After test reading, each device was photographed to allow a control quality process. Finally, sensitivity and specificity estimates were precisely assessed for each phenotype. Thus, we conclude that our study findings are robust.

Based on the HPLC reference method, Kanter et al. found similar results in seventy-one US subjects aged 6 months to 72 years, diagnosed by HPLC for HbAA, HbAC, and SCD phenotypes [13] with 99% sensitivity and 99% specificity. We noted a 2% specificity differential for HbAC phenotype in their study [13], compared to our results in Mali.

Testing 139 blood samples issued from a pediatric and adult mixed population in USA, MacGann et al. found a sensitivity of 98.3% to 100% and specificity of 92.5–100% to detect the presence of HbA, HbS, and HbC compared to CE as a standard [15]: thus, a lower sensitivity and specificity rates in HbAA persons, with a discrepancy equal to − 1·7% and − 6·0%, respectively, and in HbCC patients. We found similar results in our study conducted in Togo. McGann et al. also described excellent inter-observer agreement [15]. Finally, two recent studies were conducted in field conditions in sub-Saharan countries. In a preliminary small sample-size study in Nigeria, it diagnosed SCD with 100% sensitivity and 98% specificity compared to HPLC as the standard in 57 adults and pediatric patients (including only one HbSS and one HbSC) [16]. In Tanzania, it diagnosed SCD with 98.1% sensitivity and 91.1% specificity compared to electrophoresis for un-experienced observers in 745 participants [17]. These results were very similar to ours.

Our study has three limitations. First, due to random variation, no case of S/β°thalassaemia was included in the cases of Bamako setting. But the principle of the test already suggests that such phenotypes cannot be recognized because their definition requires the determination of minor fractions of hemoglobin (HbF and HbA2), red cell indices, level of iron stores of body, and sometimes family and genetic studies [15]. Second, we did not assess test accuracy during the neonatal period, for logistical reasons but the accuracy assessment of the Sickle SCAN® test on fetal Hb forms remains of interest. Third, we were not able to estimate negative and positive predictive values for a given screening strategy; these performance measures will be further addressed in a separate study to be conducted in new-born. Finally, we were not able to provide more details on the cost of the current diagnosis methods used in Togo and Mali that are more complex to assess with both direct and indirect costs. In our study, the test price ranged from US$5 to US$6 (provided free of cost by manufacturer and excluding taxes in 2016), this relatively affordable test received positive feedback from health professionals regarding its ease of use and its speed and convenience. Elsewhere, the commercial costs alone can run to $5–10 per test in resource-limited setting and should more affordable with its regular use [22].

## Conclusion

Thus, we conclude that the Sickle SCAN® test demonstrates excellent to perfect (HbAS, HbAC, HbSS, HbSC) or perfect (HbCC and HbAA) diagnostic accuracy, under both laboratory and field conditions. This rapid test demonstrates outstanding sensitivity and specificity when evaluated against existing gold standards. In addition, this test allows instrument- and electricity-free visual diagnostics, and requires minimal training to be performed. This rapid test is cheaper, easier to implement, and logistically more convenient than the current standard diagnostic methods in use in West Africa.

Further studies are now crucial to assess the predictive value indicators and the rate of true positive/negative among people receiving a Sickle SCAN® test result. More specially, there is a need to assess this POC device to detect SCD in the neonatal period, and fully measure the interaction with fetal hemoglobin, to inform the implementation of a neonatal screening strategy that could change the future of patients born with SCD.

While more research is needed to study the performances of the rapid test in the neonatal population, this study henceforth adds important new data to the growing evidence that this POC test could be used as first-line in a screening strategy to eliminate healthy subjects first before confirming remaining cases or indeterminate with a standard reference test. It has the potential to significantly impact the delay of diagnosis and improve the access to early care in patients living with SCD in resource-limited settings [13].

## Abbreviations

aOR: Adjusted odds ratio
AUC: Area under the curve
CE: Capillary electrophoresis
CI: Confidence intervals
CRF: Case Report Form
Hb: Hemoglobin
HPLC: High Performance Liquid Chromatography
IEF: Isoelectric focusing
POC: Point-Of-Care
SCD: Sickle cell disease
